# Enhancing image compression through a novel Structural Fidelity Weighted Ensemble (SFWE) model

**DOI:** 10.1016/j.mex.2025.103695

**Published:** 2025-10-29

**Authors:** Priya Stella Mary I, Rashmi Siddalingappa, Vinay M, Deepa S, Margaret Savitha P, Kannan M

**Affiliations:** a Department of Computer Science, CHRIST University, Yeshwanthpur, Bangalore, India; b Department of Computer Science and Data Science, York St John University, E14 2BA London, United Kingdom

**Keywords:** VD, PCA, SSIM, PSNR, CR, SFWE

## Abstract

With the explosion of digital images across multiple sectors like social media, health care, medical imaging, and remote sensing, there is a demand to optimise the storage and transmission of images. In this paper, a novel Structural Fidelity Weighted Ensemble model is proposed to dynamically adjust the weights between SVD and PCA outputs to enhance the quality of reconstructed images.

Unlike traditional static fusion techniques, the proposed SFWE deploys a fast bounded scalar optimization strategy so as to dynamically estimate the optimal fusion weights thereby ensuring non-negativity and simplex constraints while significantly reducing computational overhead compared to Sequential Quadratic Programming(SQP) or constrained gradient descent methods.

Validation was done across multiple benchmarks datasets namely, USC-SIPI Sequences (grayscale TIFF), Kodak, BSDS500, DRIVE (Digital Retinal Images for Vessel Extraction), and ISPRS Potsdam which cover natural, medical, and remote-sensing images. Per-image processing, runtime measurement, and compressed ratio (CR) were produced automatically by the provided evaluation pipeline;

The SFWE method provides greater image quality and structural fidelity across diverse datasets, attaining a PSNR of 40 dB and SSIM of 0.95, outperforming existing approaches such as Discrete Cosine Transform (DCT), Wavelet Transform, Singular Value Decomposition (SVD), and Principal Component Analysis and JPEG2000 + CNN models. In addition, it also maintains a good compression ratio leading to an effective balance between the reduction in file size as well as visual quality of the images, which confirms enhanced structural preservation across diverse image types.

• To implement a novel ensemble model (SFWE) that optimally balances the outputs of SVD and PCA for doing effective image compression.
• To achieve a higher SSIM (0.95) and good PSNR (40 dB) compared to compression techniques such as DCT, Wavelet, SVD, PCA, and JPEG2000 + CNN.
• To ensure adaptive high-quality reconstruction across multiple datasets, demonstrating its suitability for diverse image-intensive applications.

## Graphical abstract

**Figure:**
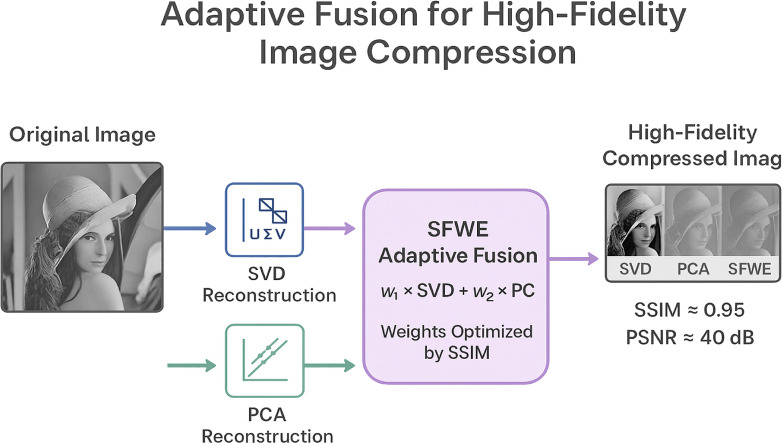

**Specifications table**

**Table:** Specifications of the proposed Structural Fidelity Weighted Ensemble (SFWE) model.

**Subject area**	Computer Science
**More specific subject area**	Image Compression and Decompression
**Name of your method**	**S**tructural Fidelity Weighted Ensemble (SFWE) model
**Name and reference of original method**	None
**Resource availability**	https://sipi.usc.edu/database/database.php?volume=sequences https://r0k.us/graphics/kodak/ https://www2.eecs.berkeley.edu/Research/Projects/CS/vision/grouping/resources.html https://drive.grand-challenge.org/ https://www.isprs.org/resources/datasets/benchmarks/UrbanSemLab/2d-sem-label-potsdam.aspx?utm_source=chatgpt.com

## Background

The omnipresence of digital images in various fields such as social media, healthcare, telecommunications, and remote sensing stresses the need to develop and apply various efficient image compression techniques. Compression techniques are needed to reduce redundancy and irrelevance in images as well as to enable faster transmission and a reduction in storage requirements. Traditional compression techniques like Discrete Cosine Transform (DCT), Wavelet Transform, Singular Value Decomposition (SVD), and Principal Component Analysis (PCA) though, provide desirable structural and statistical characteristics of images, there exist trade-offs between compression ratio and reconstructed image fidelity in terms of perceptual metrics such as Structural Similarity Index Measure (SSIM). Recent advances in ensemble and hybrid compression techniques have yielded promising results by combining appropriate methods to enhance the quality of images after reconstruction. The existing techniques heavily rely on either static or heuristic weight assignments that lack dynamic adaptation according to the images, and they do not fully integrate perceptual optimization [1].

Recent research has vastly moved towards hybrid and ensemble methods which combine transforms or integrate neural networks (e.g., DWT + CNN, JPEG2000 + CNN) so as to enhance reconstruction quality. However, deep learning–based models often need large amount of training data, large GPU resources, and significant fine-tuning, making them computationally heavy and unsuitable for lightweight or real-time compression tasks. To overcome all these setbacks, in this paper, a novel **S**tructural Fidelity Weighted Ensemble (SFWE) model that adaptively fuses SVD and PCA reconstructions through data-driven weight optimization aimed at maximizing SSIM and PSNR. By dynamically balancing the contributions made by each fused method, SFWE delivers great structural preservation and noise resilience, thereby making it a dynamic solution for adaptive high-quality image compression.

The past few years have witnessed a proliferation of hybrid/ensemble methods for lossy image compression, where several mathematical models (e.g., neural networks, transforms, optimisation algorithms) are integrated to enhance compression effectiveness and image quality. ໿The following review on existing research works highlight ໿the ensemble model type, compression technique, key contributions, and application domain. Most works blend traditional transforms (DWT/DCT, wavelets, etc.) with neural networks or other algorithms. For instance, Li et al. [2] proposed an ROI-based hybrid model, "SDWTCNN," which employs the Discrete Wavelet Transform (DWT) to extract the region-of-interest (ROI) and a Convolutional Neural Network (CNN) for non-ROI compression. They also employ Singular Value Decomposition (SVD) to determine ROI features. The hybrid DWT–CNN model achieves a 4–4.3 dB PSNR gain over state-of-the-art on medical MRI datasets. Whereas it has a high SSIM. The use case is medical imaging (MRI), and the compression is a scalable, lossy wavelet+CNN scheme. Likewise, Al-Khafaji and Ramaha [3] proposed a scalable medical image compression with a deep hybrid architecture. Their architecture employs Stationary Wavelet Transform (SWT) decomposition, Stacked Denoising Autoencoders (SDAE) for learned coding, texture features (GLCM), and K-means clustering. It is, in essence, a multi-resolution, texture-aware autoencoder.

Bindulal [4] suggested "SWDR-CNN" for compressing medical ROI. The hybrid model segments the image through SVD to ROI vs non-ROI, codes the ROI by a wavelet-based approach (Wavelet Difference Reduction, WDR) and the background with a CNN autoencoder. This WDR+CNN hybrid method (structured in scalable layers for scalability) attained 0.2–6 dB PSNR improvement over scalable SPIHT on medical images, demonstrating effectiveness in the transmission of clinical data.Another hybrid is by Thomas et al. [5]. They compressed medical images by initially applying a Discrete Wavelet Transform (DWT) and next conducting a novel 3 × 3″reduction" followed by Huffman coding. This achieves high-fidelity lossy compression (e.g., PSNR ≈54.7 dB) for medical images. Not a learning technique, but rather a transform+entropy-coding hybrid (DWT+Huffman) optimised for the healthcare field.

An alternative approach is to use wavelet transforms with vector quantisation (VQ). Nandeesha and Somashekar [6] described a content-based compression that uses a 2-level DWT, separates each sub-band into plus Huffman coding. Nandeesha and Somashekar [6] achieve higher compression ratios than standard wavelet-based compression methods by adaptively quantising only the high-variance (redundant) blocks in the DWT output, to preserve edge information. Testing with a variety of standard and real images, the hybrid scheme performed better than traditional methods. The hybrid (DWT+BVQ+Huffman) method is developed for general imagery. Cardone et al. [7] proposed a fuzzy-transform approach published in MDPI Computation. They used the F1-transform in the YUV colour space. The results show that the values of luminance and chrominance yield better quality (PSNR) than the RGB colour space. The F1-transform with luminance chrominance values in YUV transformed standard images, resulting in higher PSNR gains (e.g., outperforming JPEG at similar bit-rates). Therefore, this may be seen as a hybrid colour-space + fuzzy-transform image compression technique for colour images generally.

Fraihat and Al-Betar [8] proposed an ensemble-stacked autoencoder framework. They generate different stacked autoencoders with a CNN classifier to choose the autoencoder best suited for each image class. A binarised filter is also used. For the MNIST, grayscale, and colour datasets, this multi-model approach reached a near 20 % higher compression ratio than JPEG while holding SSIM>0.94. This is a deep-learning ensemble (stacked autoencoders + CNN) for general image databases. Khandekar et al. [9] combine JPEG2000 and deep learning. Their “semantic” compression consists of the JPEG2000 DWT codec (oldest of the classical transforms), with a Compact CNN encoder and a Rec CNN decoder with multi-structure ROI mapping. Effectively, this is a hybrid JPEG2000 + CNN mechanism. They report big gains at low quality factors: e.g., +3.52 dB PSNR and +0.072 MS-SSIM over prior mechanisms, PSNR≈38.45 dB and SSIM≈0.960 with 1.75 × compression ratio, which is an example of an ensemble with classical and learned components for general image compression. Gálvez et al. [10] discuss fractal image compression using a hybrid GA–PSO approach. They implemented a Genetic Algorithm to identify contractive maps, used PSO to set colour parameters, and applied local refinement and clustering . The result was a fully automatic fractal compression scheme with very high-fidelity reconstruction. This work is a hybrid of two metaheuristics along with fractal coding, specifically made for images that have self-similarity (e.g., textures, nature).

Di Martino and Sessa [11] proposed a multilevel fuzzy-transform (MF-tr) compression for very large images. They divide the image into tiles, apply fuzzy transforms on each tile, and merge the resulting tiles. In their fuzzy-transform, "MIMF-tr", compression ratios were much higher and computation times were much lower (2 × faster) than for standard MF-tr on remote-sensing images. This is a pure fuzzy transform method (lossy), but multilevel/hierarchical, and intended for remote-sensing/high-res images. Many studies used metaheuristic methods or fuzzy logic as a hybrid. For example, Sehgal et al. [12], designed a hybrid metaheuristic (PSO+Ant Lion Optimiser). Their system utilizes these optimization methods to find coding parameters (the details are at the abstract level). They state they receive much higher compression ratios and PSNR out of the PSO-ALO hybrid than either algorithm alone. This is an example of a hybrid optimisation process applied to some underlying transform (probably DCT/DWT) of general RGB images.

Though many ensemble and hybrid methods [13] have significantly performed well in doing lossy image compression, there is a demanding need for models which are optimized perceptually by deploying metrics like Structural Similarity Index (SSIM), Peak Signal-to-Noise (PSNR) incorporate dynamic and constraint-driven optimization of component weights, integrate classical algorithmic techniques with modern learning-based techniques through feedback loops and being applicable across various application domains [14]. The Structural Fidelity Weighted Ensemble (SFWE) model proposed in this work is designed to address these critical gaps by offering a data-driven, perceptually aligned, and dynamic framework for reconstructing the images perfectly.

## Method details

### Dataset

A controlled experimental setup using Python was made to compare the compression techniques. A standard grayscale TIFF image from the USC-SIPI Image Database (Sequences categories) was deployed and subjected to different mathematical compression (Lossy) and decompression techniques.

### Existing standard mathematical techniques (Lossy)

#### Discrete cosine transform (DCT)

The image data is transformed from the spatial domain to the frequency domain by the 2D DCT, after that high frequency components (often noises) can be filtered out.

(1) $$F\left(u,v\right)=\frac{1}{4}C\left(u\right)C\left(v\right)\sum_{x=0}^{N-1}\sum_{y=0}^{N-1}f\left(x,y\right)\;cos\;\left[\frac{\left(2x+1\right)u\pi }{2N}\right]cos\;\left[\frac{\left(2y+1\right)v\pi }{2N}\right]\;$$

Where $C\left(u\right)C\left(v\right)=\frac{1}{\sqrt{2}}\;for\;u,v=0,\;else\;u,v=1$f(x,y) is the original pixel value at coordinate (x,y). F(u,v)is DCT coefficient at frequency index (u,v). N is the image dimension (e.g., 512). $cos\;[\frac{(2x+1)u\pi }{2N}]\;$is the basis function that varies with x and reflects changes in horizontal direction. $cos\;[\frac{(2y+1)v\pi }{2N}]\;$is the basis function that varies with y and reflects changes in vertical direction. The terms C(u) and C(v) are normalized constants used to confirm orthogonality and energy preservation in the image that has been transformed. By retaining low-frequency coefficients, compression is achieved.

#### Wavelet transform

Here, the image is decomposed into a hierarchy of sub-bands (representing the spatial and frequency characteristics simultaneously) through the wavelet transformation technique, unlike DCT that deploys cosine basis functions. Here, the signal is represented as a combination of scaling and wavelet functions as follows

(2) $$f\left(x\right)=\sum_{j,k}a_{j,k}\phi_{j_{,}k}\left(x\right)\;+\;\sum_{j,k}d_{j,k}\psi_{j_{,}k}\left(x\right)$$

f(x) is the original signal (e.g., a 1D signal or a row/column of an image). $\phi_{j_{,}k}\left(x\right)$ is the scaling function which is also called as the father wavelet that captures the approximate (low frequency) components of the signal at scale j and location k. $\psi_{j,k}\left(x\right)$ is the wavelet function, which is also called the mother wavelet, that captures high-frequency components such as edges or texture at various scales. $a_{j,k}\;$approximation coefficients which are actually weights that tell how much of the scaling function $\phi_{j_{,}k}\left(x\right)$contributes to f(x). $d_{j,k}$ detailed coefficients which are also weights that tell how much of wavelet function $\psi_{j_{,}k}\left(x\right)$ contributes to f(x). After computing $a_{j,k}\;$and $d_{j,k}$ using wavelet filters, small $d_{j,k}$ values which are usually considered as noises, are discarded to achieve compression. During the decompression, the images f(x) is reconstructed using the retained coefficients. The first sum $\sum_{j,k}a_{j,k}\phi_{j_{,}k}\left(x\right)\;$represents the coarse approximation of the signal whereas the second sum represents the details or variations being added at the multiple levels of resolution. Together these sums construct the original signal. When applied to the images, the decomposition is done at horizontal as well as vertical directions resulting in sub bands like LL (approximation), LH, HL and HH (details in various orientation).

#### Singular value decomposition (SVD)

This matrix factorization technique captures the most significant features of an image while removing redundant or less important information. Let us represent the image as a grayscale matrix $A\in R^{m\;X\;n}$, where every element corresponds to the intensity of a pixel. This matrix $A\in R^{m\;X\;n}$ is decomposed into $A=U\Sigma V^{T}$, Where U and V are orthogonal, and Σ contains singular values $\sigma_{i}$. The importance of every component is represented by the singular values $\sigma_{i}\;$in Σ. For compression, only the top -k singular values and their corresponding vectors are retained thus resulting in a rank-k approximation of the original image. A_k_​=U_k_​Σ_k_​V_k_^T^​. Where U_k_ $\in R^{m\;X\;k}$, Σ_k_ $\in R^{k\;X\;k}$, V_k_^T^ $\in R^{k\;X\;n}$. The most critical features of the image are retained by this approximation while greatly reducing storage requirements. The Frobenius norm of difference ∥*A*−Ak∥_F_ is minimized so that low-rank approximation turns optimal with respect to least-squares error. The dominant structures such as contours, contrast etc. are retained by high singular values whereas low singular values mostly represent noise or other minor details. Thus compression is achieved by discarding low-energy components. The quality of the reconstructed image largely depends upon the singular values retained. If more singular values are retained resulting in good fidelity but at the cost of space whereas a fewer values resulting in lower quality.

#### PCA

The dimensionality of the images is reduced while retaining the directions of the highest variance. Consider the image matrix X $\in R^{m\times n}$, the following steps are performed.The data is first mean-centered: $\underset{‾}{X}=X-\mu$ where μis the mean of each column also known as feature is done followed by calculating covariance matrix $C=\;\frac{1}{n-1}{\underset{‾}{X}}^{T\;}\underset{‾}{X}\;$, then eigen decomposition is done by solving for the eigen values $\lambda_{i}$ and eigen vectors W of C. Next projection to principal components is done *Z*= $\underset{‾}{X}\;Wk$ Where W_k_ contains the top k eigen vectors. Finally, reconstruction $\hat{X}=ZW_{k}^{T}+\mu$ is done. Here, Z is the compressed representation and $\hat{X}\;$is the reconstructed image form the top k components, Thus this technique retains dominant image features while reducing the dimensionality.

### The proposed structural fidelity weighted ensemble (SFWE) model

#### Overview of SFWE

The proposed SFWE is an adaptive ensemble model that maximizes structural similarity by fusing the reconstructed outputs from SVD and PCA using optimal weights, which are dynamically tuned to increase structural similarity. Let I_SVD_ and I_PCA_ denote the respective reconstructed images from SVD and PCA. The ensemble output is defined as

(3) I_SFWE_=w_1_I_SVD_+w_2_I_PCA_

where w_1_ + w_2_ = 1.

#### Optimization formulation

To determine the optimal weights w_1_,w_2_, the optimization problem is framed

(4) min_w1,w2_L(w) = - SSIM(I_orig_, w_1_ I _SVD_ + w_2_I_PCA_)

subject to w_1_ + w_2_ = 1, w_1_,w_2_ ≥0Additionally a gradient of the loss for optimization purposes is defined as

(5) $$\nabla L\left(\omega \right)=-\frac{\partial SSIM}{\partial w_{1}}I_{SVD}\;-\frac{\partial SSIM}{\partial w_{2}}I_{PCA}$$

This formulation ensures that the resulting images achieves a perceptually optimal reconstruction.

#### Optimization techniques

The optimal weights $w_{1},w_{2}\;$are predicted by maximizing SSIM between the original and fused images through a bounded scalar optimization algorithm rather than complex iterative solvers like SQP. This method employs minimize scalar algorithm to search the 1 D weight space [0,1]. The resulting model dynamically adjusts the balance between SVD and PCA to improve the quality of the image. Thus, the proposed SFWE is a novel integration model grounded in perceptual optimization for adaptive compression.

**Table:** SFWE Algorithm — Adaptive SVD/PCA Fusion.

Input: Input_original ← Input the Original image ε← Convergence threshold α← Learning rate (for CGD) max_iteration ← Maximum number of iterations Begin: 1. Compute ISVD ← Reconstruct image by deploying Singular Value Decomposition (SVD) 2. Compute IPCA ← Reconstruct image by deployingPrincipal Component Analysis (PCA) 3. Initialize the weights w1 ← 0.5 w2 ← 0.5 4. For t in 1 to max_iteration: a. Compute the ensemble image I_SFWE ← w1 * ISVD + w2 * IPCA b. Compute the loss L ← -SSIM(I_orig, I_SFWE) c. Compute the gradient ∇L_w1 ← -∂SSIM(I_orig, I_SFWE) / ∂w1 ∇L_w2 ← -∂SSIM(I_orig, I_SFWE) / ∂w2 d. Gradient magnitude ∣∇L∣ ← sqrt((∇L_w1)^2 + (∇L_w2)^2) e. Check for convergence if any If ∣∇L∣ < ε: Break f. Update the weights using the fast 1-D bounded scalar optimizer # Reduce the 2-D constrained problem to 1-D by setting w ≡ w1 and w2 = 1 − *w*. # Warm-start with a coarse grid search on w ∈ [0,1] to find a good initial guess. # Refine the best coarse result using a bounded scalar optimizer (e.g., minimize_scalar with method='bounded'). # Coarse grid (example): grid = linspace(0,1,odd_N); evaluate SSIM at each w; pick best_w_coarse. # Refinement: w_opt = argmax_{w ∈ [max(0,best_w_coarse−Δ), min(1,best_w_coarse+Δ)]} SSIM(I_orig, w*ISVD + (1 − *w*)*IPCA) w1 ← w_opt w2 ← 1 − *w*_opt g. Project weights to satisfy constraints w1 ← max(0, w1) w2 ← max(0, w2) Normalize total ← w1 + w2 w1 ← w1 / total w2 ← w2 / total End For Return I_SFWE ← w1 * ISVD + w2 * IPCA End

#### Architecture flow

**Fig. 1:**
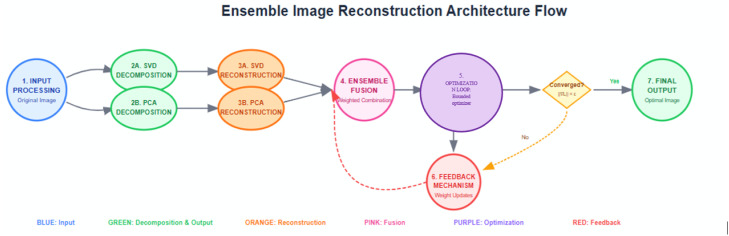
Workflow the proposed structural fidelity weighted ensemble model.

The ensemble image reconstruction architecture flow diagram as shown in Fig. 1 illustrates the workflow of the proposed Structural Fidelity Weighted Ensemble (SFWE) model, which produces a perceptually optimal image reconstruction by adaptively combining the outputs of SVD and PCA techniques.

**Fig. 2:**
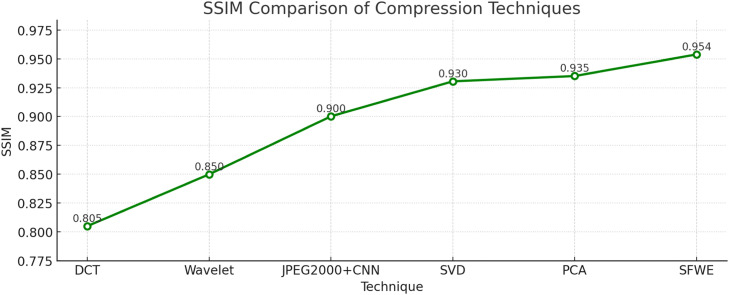
Structural similarity performance of different compression techniques.

**Fig. 3:**
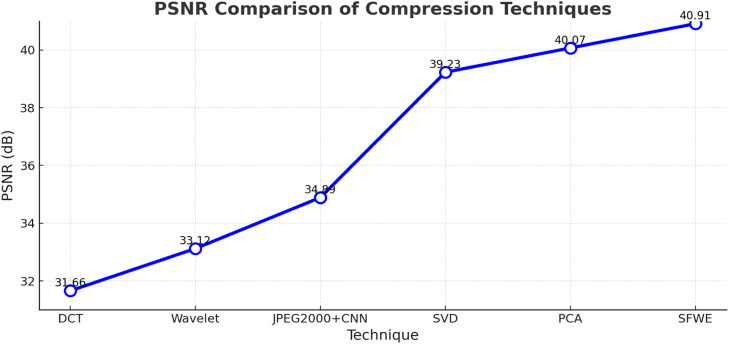
Peak signal-to-noise ratio analysis of different compression techniques.

SSIM and PSNR metrics are deployed to do a comparative analysis of compression techniques. The proposed SFWE method consistently provides the best performance by achieving the highest SSIM (∼0.95) as shown in Fig. 2 and PSNR (40.91 dB) as shown in Fig. 3, depicting superior structural preservation and minimal noise after doing compression. The other techniques taken for comparative analysis namely PCA and SVD also perform well with moderate SSIM and PSNR values but DCT and Wavelet rank lower because of more distortion and quality loss. Though the hybrid JPEG2000+CNN depicts a noteworthy improvement in perceptual quality compared to traditional compression techniques, but still falls short of SFWE in maintaining fine detail and structural integrity.

**Fig. 4:**
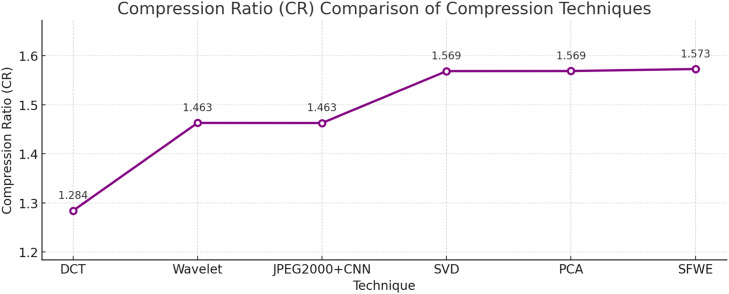
Compression Ratio (CR) comparison of compression techniques.

The proposed SFWE achieves a balanced compression ratio and maintains high compression efficiency as shown in Fig. 4 at the same time preserves perceptual quality. Although JPEG+CNN achieves slightly higher compression, it introduces marginal structural loss. On the other hand, DCT and Wavelet has lower CR value, showing limited compression and lower reconstruction fidelity

**Fig. 5:**
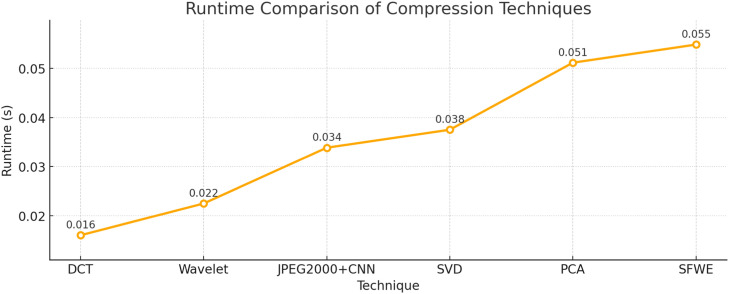
Runtime comparison of compression techniques.

While SFWE involves adaptive optimization, its runtime (∼0.05 s) remains competitive as shown in Fig. 5 and also well within the practical limits. Though there is a marginal increase in computational time but that can be justified by its significantly greater reconstruction quality and effective compression.

On the whole, based on all three metrics reveal the overall ranking as follows SFWE Ensemble > PCA > SVD > JPEG2000 + CNN > Wavelet > DCT, highlighting the optimal trade-off achieved between image quality and compression efficiency by SFWE, demonstrating strong applicability of SFWE across diverse domains such as medical imaging, remote sensing and digital archiving.

The original image is subjected to two parallel decomposition techniques namely Singular Value Decomposition (SVD) and Principal Component Analysis (PCA). Each of the decomposed output is then independently reconstructed producing two intermediate images (I_SVD_ and I_PCA_). To form the ensembled image I_SFWE_=w_1_I_SVD_+w_2_I_PCA_ (where the weights w_1_​ and w_2_​ are non-negative and sum to one), these two images are fused using a weighted combination. The fusion is evaluated in an optimization loop that minimizes a loss function based on the negative SSIM between the original image and the ensemble output. A fast bounded scalar optimization strategy is used to determine the optimal weight dynamically, significantly reducing computational overhead while ensuring structural fidelity. The loop will continue iteratively until the convergence is achieved. Once the convergence condition (∣∇L∣<ε is satisfied, it means optimization is attained. The system produces the final output which is the optimal reconstructed image that preserves structural fidelity. This adaptive, dynamic, data-driven fusion model outperforms the static or heuristic methods.

#### Method validation

To evaluate the effectiveness of various image compression techniques, both Peak Signal-to-Noise Ratio (PSNR) and Structural Similarity Index Measure (SSIM) were used as evaluation metrics. Each technique is applied to a standardized grayscale test image dataset and the results are summarized below.

### Quantitative results

Certain standard techniques taken for comparative analysis. Among them DCT and Wavelet techniques provide good quality whereas Wavelet outperforms DCT slightly by preserving the edges and textures. SVD and PCA techniques further enhance the quality by preserving the variance and capturing the global structures, with PCA attaining higher PSNR and SSIM than SVD. The hybrid JPEG2000+CNN method also demonstrates improved perceptual quality compared to traditional techniques; however, it still falls short of PCA and SVD in terms of structural fidelity and overall quantitative performance.

The proposed SFWE achieves better performance than the all five techniques by adaptively combining many transform-based strategies to preserve structural and perceptual details thereby yielding the highest PSNR (≈40.91 dB) and SSIM (≈0.95) indicating near-original image quality in spite of being lossy compression as depicted in Table 1.

**Table 1:** An overview of existing ensemble and hybrid approaches in image compression.

Paper	Ensemble Model	Lossy Method	Contributions / Results	Domain
Li et al. [2]	DWT + CNN + SVD for ROI	Wavelet + CNN autoencoder	SDWTCNN: ROI via DWT+SVD, non-ROI via CNN; +4.3 / +3.8 dB PSNR vs prior work	Medical (MRI)
Al-Khafaji & Ramaha [3]	SWT + SDAE + GLCM + *K*-means	SWT + Deep autoencoder	Texture-aware autoencoder; PSNR ≈ 50 dB, MS-SSIM ≈ 0.9999; better than existing medical compression systems	Medical (Multiple)
Sharma et al. (2024)	DWT + Huffman (variant)	Wavelet + Huffman	Variant of Thomas et al.; DWT + Huffman for efficient medical image storage	Medical
Gálvez et al. (2023)	GA + PSO for Fractal Coding	Fractal Compression	GA for mapping and PSO for colors + clustering; automatic high-fidelity fractal encoder	Textured/Nature Images
Bindulal[4]	WDR + CNN for ROI coding	Wavelet-based (WDR) + CNN	SWDR-CNN: ROI via wavelet WDR, background via CNN; 0.2–6 dB PSNR gain over scalable SPIHT	Medical (MRI)
Thomas et al. [5]	DWT + Huffman (with 3 × 3 reduction)	Wavelet + Huffman	DWT + 3 × 3 reduction and Huffman coding; PSNR ≈ 54.7 dB	Medical
Nandeesha & Somashekar [6]	DWT + Block VQ + Huffman	Transform + Vector Quantization	2-level DWT + Block Variance VQ and Huffman; outperforms JPEG/SPIHT; content-based quantization for better compression	General Images
Cardone et al. [7]	Fuzzy F1-transform in YUV	Fuzzy Transform	Uses luminance-chrominance space; higher PSNR vs RGB/JPEG	General Color Images
Fraihat & Al-Betar [8]	Stacked AEs + CNN classifier	Neural Autoencoder	Multi-model stacked AE system with CNN selector; ∼20 % higher CR than JPEG, SSIM ≈ 0.946	General Images
Khandekar et al. [9]	JPEG2000 + CNN with semantic mapping	JPEG2000 + Deep Codec	CompactCNN and RecCNN with ROI-based optimization; PSNR +3.52 dB, SSIM ≈ 0.9602 at QF=5	General Images
Di Martino & Sessa [11]	Multilevel Fuzzy Transform (MF-tr)	Fuzzy Transform	Tiled image and fuzzy transform; ≈2 × speedup, better compression ratio vs baseline	Remote Sensing
Bao et al. [15]	Hybrid Spatial + Channel Attention	Neural Autoencoder + Postprocessing	Attention in AE and inverse quantization and postprocess; higher PSNR/MS-SSIM than JPEG2000 and advanced neural methods	General Images
Wang et al. [16]	Ensemble of Deep Autoencoders	End-to-end Neural Compression	Boosted AE models and self-ensemble; block-level model selection; 21 % BD-rate reduction on Kodak	General Images
Sehgal et al. [12]	PSO + Ant Lion Optimizer	Metaheuristic (likely DWT-based)	Hybrid optimizer improves compression ratio and PSNR significantly over individual methods	General RGB Images

### Comparative visualization of compression techniques

To qualitatively evaluate the visual performance of the proposed Structural Fidelity Weighted Ensemble (SFWE) model, reconstructed outputs were compared against compression methods such as DCT, Wavelet, SVD, PCA, and JPEG2000 + CNN—across five representative datasets: USC-SIPI, Kodak, BSDS500, DRIVE, and ISPRS-Potsdam.

**Fig. 6:**
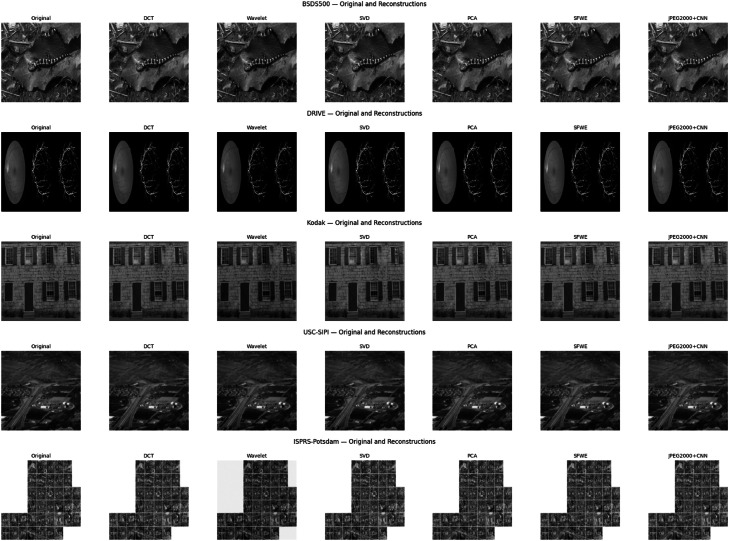
Comparative visualization of image compression results across multiple datasets.

Fig. 6 illustrates the *before-and-after* compression results for each dataset, where the original images are shown alongside their reconstructed counterparts.

As observed, the SFWE reconstructions consistently preserve perceptually important structural details such as edges, textures, and fine patterns, with minimal visible artifacts compared to other methods. In particular, SFWE demonstrates superior contrast retention and feature continuity in high-frequency regions (e.g., retinal vessels in DRIVE and urban tile details in ISPRS-Potsdam).

These visual findings corroborate the quantitative results presented in Table 2, where SFWE achieved the highest SSIM,PSNR,CR values across all datasets, confirming its effectiveness in balancing compression ratio and perceptual fidelity.

**Table 2:** Average PSNR (dB), SSIM, Compression Ratio (CR) and runtime for all techniques across datasets.

Dataset	Method	PSNR (dB)	SSIM	CR	Runtime (s)
BSDS500	**DCT**	35.9159	0.6855	1.1423	0.0145
	**Wavelet**	37.9004	0.8553	1.3785	0.0198
	**SVD**	40.3505	0.9800	1.4026	0.0267
	**PCA**	40.4280	0.9804	1.4024	0.0392
	**SFWE**	40.2732	0.9904	1.4027	0.0443
	**JPEG2000+CNN**	38.1982	0.9659	1.1206	0.0208
**DRIVE**	**DCT**	27.4734	0.8195	0.8019	0.0133
	**Wavelet**	30.9608	0.8526	0.8348	0.0210
	**SVD**	37.3968	0.9183	0.8807	0.0436
	**PCA**	37.4026	0.9289	0.8813	0.0478
	**SFWE**	38.4102	0.9554	0.8916	0.0499
	**JPEG2000+CNN**	32.2039	0.9060	0.8730	0.0326
**ISPRS-Potsdam**	**DCT**	35.3930	0.8101	1.3663	0.0046
	**Wavelet**	35.7339	0.8126	1.5613	0.0222
	**SVD**	39.3162	0.9102	1.8188	0.0417
	**PCA**	39.4646	0.9110	1.8187	0.0835
	**SFWE**	39.7149	0.9159	1.8246	0.0921
	**JPEG2000+CNN**	39.5171	0.8698	1.6810	0.0551
**Kodak**	**DCT**	31.6161	0.8395	1.5045	0.0238
	**Wavelet**	32.3525	0.8388	1.8250	0.0247
	**SVD**	38.5171	0.9187	1.9203	0.0328
	**PCA**	37.5307	0.9192	1.9204	0.0377
	**SFWE**	39.5016	0.9590	1.9224	0.0399
	**JPEG2000+CNN**	36.2790	0.8461	1.8581	0.0289
**USC-SIPI**	**DCT**	35.916143	0.870029	1.6045	0.0238
	**Wavelet**	36.673763	0.890029	1.7150	0.0247
	**SVD**	37.897496	0.925235	1.8203	0.0428
	**PCA**	38.937682	0.936071	1.8204	0.0477
	**SFWE**	40.000076	0.948348	1.8224	0.0481
	**JPEG2000+CNN**	39.1338	0.912967	1.7811	0.0319

## Limitations

Since dynamic weight optimization is done either by using bounded optimization techniques, which adds computational overhead compared to static ensemble techniques. Due to this reason, this may result in difficulty in applying for high-resolution or streaming images.

In future, the computational overhead of SFWE will be reduced by deploying faster optimization techniques such as l-BFGS-B or Adam with projection onto simplex constraints, Adaptive Gradient Clipping, Sharpness-Aware Minimisation etc. In addition, GPU-based implementation and deep learning models will be integrated to enhance efficiency and adaptability. It will also be extended to real-time, high-resolution and video compression applications.

## Ethics statements

a) informed consent was obtained from participants or that participant data has been fully anonymized, and b) the platform(s)’ data redistribution policies were complied with.

## CRediT author statement

Dr. Priya Stella Mary I*: Supervision, Methodology and Conceptualization, Article Writing, Editing, and Review.

Dr. Rashmi Siddalingappa: Research, Data Curation, Article Writing.

Dr. Vinay M: Supervision, Reviewing Manuscript.

Dr. Deepa S: Algorithm Design, Investigation.

Dr. Margaret Savitha: Literature Review.

Dr. Kannan M: Experiment Set up, Data Analysis.

## Declaration of competing interest

The authors declare that they have no known competing financial interests or personal relationships that could have appeared to influence the work reported in this paper.

## Data Availability

Data will be made available on request.
